# Research advances in cGAS–stimulator of interferon genes pathway and central nervous system diseases: Focus on new therapeutic approaches

**DOI:** 10.3389/fnmol.2022.1050837

**Published:** 2022-12-22

**Authors:** Jiao Ding, Yijie Dai, Jiahui Zhu, Xuemei Fan, Hao Zhang, Bo Tang

**Affiliations:** ^1^The Fourth School of Clinical Medicine, Zhejiang Chinese Medical University, Hangzhou, China; ^2^Department of Neurology, The Affiliated Hangzhou First People’s Hospital, Zhejiang University School of Medicine, Hangzhou, China

**Keywords:** cGAS, cGAMP, STING, innate immune system, autophagy, neuroinflammation, neurodegeneration

## Abstract

Cyclic GMP–AMP synthase (cGAS), a crucial innate immune sensor, recognizes cytosolic DNA and induces stimulator of interferon genes (STING) to produce type I interferon and other proinflammatory cytokines, thereby mediating innate immune signaling. The cGAS–STING pathway is involved in the regulation of infectious diseases, anti-tumor immunity, and autoimmune diseases; in addition, it plays a key role in the development of central nervous system (CNS) diseases. Therapeutics targeting the modulation of cGAS–STING have promising clinical applications. Here, we summarize the cGAS–STING signaling mechanism and the recent research on its role in CNS diseases.

## 1. Introduction

A cellular cytosolic double-stranded DNA (dsDNA) sensor known as the cyclic GMP–AMP synthase (cGAS)–stimulator of interferon genes (STING) pathway can activate the innate immune system, which can then react to conditions such as infection, inflammation, and malignancy (Jiang et al., 2020). cGAS is an innate immunological sensor that detects two main categories of cytosolic dsDNA—pathogen-derived DNA and self-DNA—including DNA from bacteria, viruses, mitochondria, micronuclei, and retroelements (Zheng et al., 2020). cGAS is activated when it binds to DNA, which results in the formation of 2′ 3′-cyclic GMP–AMP (cGAMP). The interferon response is triggered by cGAMP, which functions as a second messenger to trigger downstream pathways to produce type I interferon (IFN-I) and other pro-inflammatory cytokines by binding to the endoplasmic reticulum protein STING (Gao et al., 2013; Wu et al., 2013; Liu et al., 2019). The cGAS–STING pathway mediates immune surveillance and has neuroprotective properties. However, the excessive engagement of this pathway can also lead to negative consequences, such as neuroinflammation and neurodegeneration, and speed up the development of illnesses (Paul et al., 2021). The etiology and pathogenesis of nervous system diseases are complex, and few effective treatment methods are available; therefore, new therapeutic targets are urgently needed. Mellor et al. demonstrated for the first time that targeting STING can successfully treat CNS disorders *In vivo*. They found that DNA nanoparticles and cyclic dinucleotides treated EAE in a STING-dependent manner which delayed the onset of EAE and alleviated the severity of this disease. This paper outlines the most updated knowledge on the cGAS–STING pathway’s mechanism in central nervous system (CNS) disorders and discusses the associated therapeutic options.

## 2. cGAS–STING pathway: Discovery, activation, and involvement in autophagy

Stimulator of interferon genes, also known as transmembrane protein 173 (TMEM173), MPYS, MITA, or ERIS, is a protein on the endoplasmic reticulum (ER) that can be activated by immune-stimulatory DNA (ISD) and initiate type I interferon responses and was identified in 2008 (Ishikawa and Barber, 2008; Zhong et al., 2008; Sun et al., 2009). Subsequently, several DNA sensors, including interferon-gamma inducible protein 16 (IFI16), were found to support STING activation (Unterholzner et al., 2010). However, STING activation could not be fully explained by these upstream components and ligands, leading to the hypothesis that unidentified upstream regulators were involved. In 2013, Dr. Chen’s research team discovered cGAS, a direct cytosolic DNA receptor. This innate immunological sensor is composed of about 522 amino acids and is a member of the nucleotidyltransferase (NTase) family (Sun et al., 2013). As mentioned above, cGAS detects cytosolic dsDNA, including pathogen-derived DNA and self-DNA, which can originate from bacteria, viruses, mitochondria, micronuclei, and retroelements (Zheng et al., 2020). Independent of sequencing, cGAS can directly identify DNA of different sizes, and human cGAS can detect DNA sequences as short as 45 bp (Zhou et al., 2018). After binding to DNA, cGAS undergoes a conformational shift that catalyzes the production of 2′3′ -cGAMP from adenosine triphosphate (ATP) and guanosine triphosphate (GTP; Gao et al., 2013). Simultaneously, the binding of DNA and cGAS triggers the production of liquid-like droplets that act as microreactors, enrichment enzymes, and reactants to encourage the production of cGAMP (Du and Chen, 2018). Numerous distinct mechanisms, including ubiquitination, acetylation, phosphorylation, and cysteinase-mediated cleavage, affect cGAS activation at the post-translational level. Through several mechanisms, which involve processes such as direct modification, removal of active sites, and protein stabilization, the alterations mentioned above control the activity of cGAS (Bao et al., 2021).

**Figure 1:**
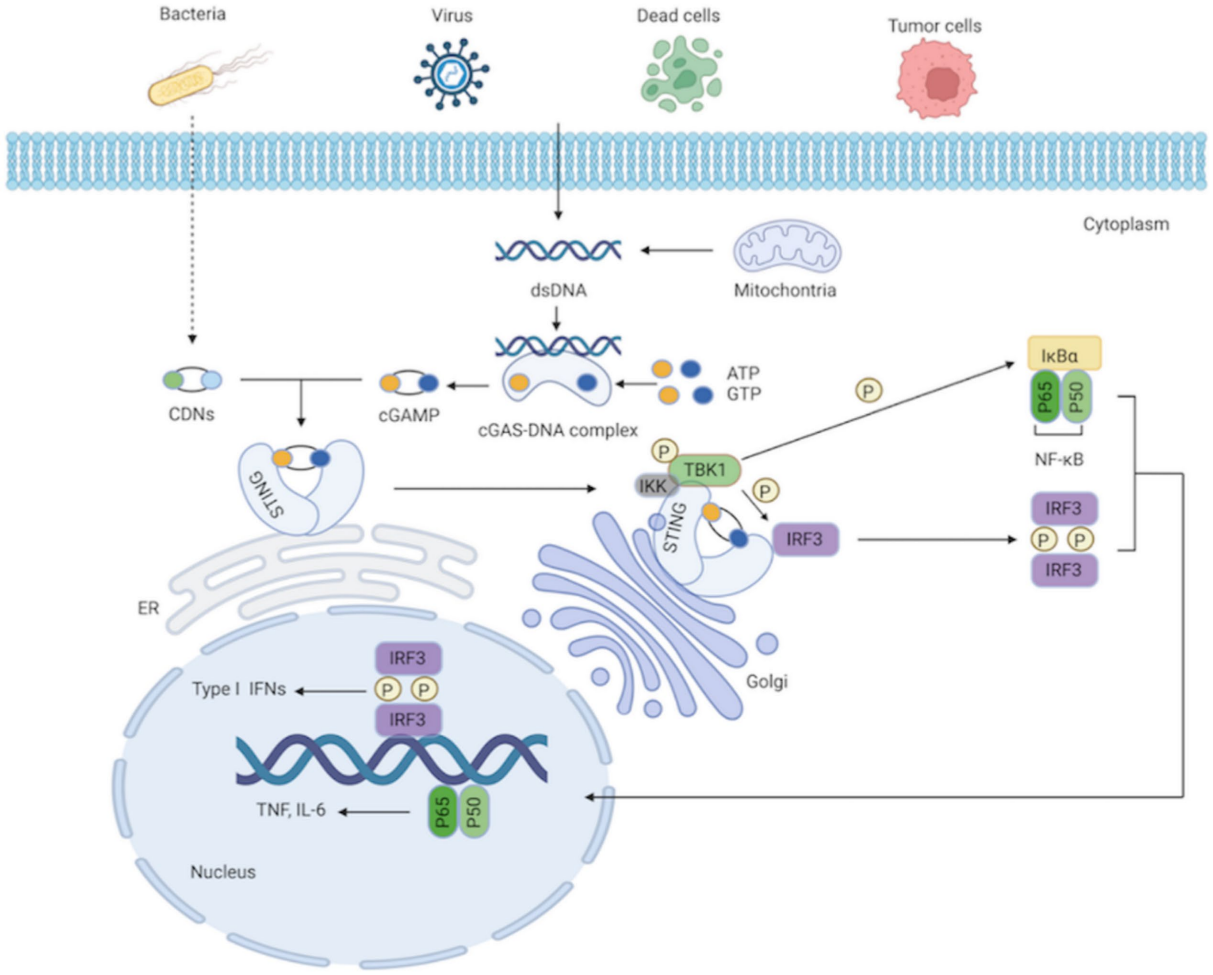
cGAS-STING signaling pathway. The cytosolic DNA sensor cGAS senses exogenous DNA from dying cells, tumor cells, viruses, bacteria, as well as endogenous DNA leaking from mitochondria. The synthesis of cGAMP by activated cGAS employing ATP and GTP as substrates, and cGAMP then activates STING by functioning as a second messenger. What’ more, the CDNs produced by bacteria can instantly activate STING. IKK and TBK1 are then recruited and activated by STING as it moves from the ER to the Golgi apparatus. IRF3 is further recruited and phosphorylated by TBK1. Afterward, phosphorylated IRF3 forms dimers, translocates to the nucleus, and functions synergistically with NF-κB, thereby resulting in the production of IFN-I and pro-inflammatory cytokines.

As a secondary messenger and activator of STING, cGAMP is a cyclic dinucleotide (CDN; Gao et al., 2013). Additionally, STING can be directly triggered by bacterial CDNs, such as cyclic di-guanylate monophosphate (c-di-GMP) and cyclic di-adenosine monophosphate (c-di-AMP; Woodward et al., 2010; Danilchanka and Mekalanos, 2013). STING is an endoplasmic reticulum receptor protein with four transmembrane domains and a cytoplasmic ligand-binding domain (LBD; Kranzusch et al., 2015). STING takes on a dimeric structure through the interaction between its transmembrane and cytoplasmic domains. The most practical component integrated with cGAMP or the CDNs is STING’s LBD. The ligand binding pocket in the LBD closes significantly after contact; this structural change generates STING tetramers, thereby activating STING (Ramanjulu et al., 2018; Shang et al., 2019). With the assistance of the Golgi apparatus, STING subsequently translocates from the ER to the perinuclear region (Tanaka and Chen, 2012). STING is palmitoylated at two cysteine residues (Cys88 and Cys91) in the Golgi apparatus, which is crucial for STING activation (Mukai et al., 2016). Modified STING attracts and activates TANK-binding kinase 1 (TBK1), which phosphorylates STING’s C-terminal domains. Interferon regulatory factor 3 (IRF3), recruited by the phosphorylated STING, is phosphorylated by TBK1 and dimerized. Dimerized IRF3 eventually translocates to the nucleus, where it stimulates the production of type I IFNs and interferon-stimulated genes (ISGs; Zheng et al., 2020). Additionally, STING can recruit and activate IκB kinase (IKK), which phosphorylates the nuclear factor-kappa B (NF-κB) inhibitor IκBα, leading to the activation of NF-κB, a heterodimer of the p65 and p50 subunits. NF-κB then translocates to the nucleus as a transcription factor and regulates the production of inflammatory cytokines, such as interleukin 6 (IL-6) and tumor necrosis factor (TNF; Ishikawa and Barber, 2008; Ishikawa et al., 2009; Zhang et al., 2013a; Motwani et al., 2019; Figure 1). After signal transduction is terminated, STING is transferred to endolysosomes for destruction (Ma et al., 2015).

Growing evidence suggests that the cGAS–STING pathway stimulates autophagy in addition to type I interferon production and inflammatory responses (Liu et al., 2018; Wan et al., 2020). Autophagy, an evolutionarily conserved stress response, regulates the breakdown and recycling of extra or hazardous cytosolic entities to protect cells from toxic protein buildup, organelle dysfunction, and pathogen invasion. It also has various effects on innate immunity (Zhang et al., 2021). Molecular chaperone-mediated autophagy, microautophagy, and macroautophagy are the three primary forms of autophagy, which differ in the way that cargo is delivered to the lysosome. Typically referred to as autophagy, macroautophagy involves the following crucial steps: initiation, nucleation or phagophore formation, membrane elongation, autophagosome formation, autophagosome fusion with the lysosome to form an autolysosome, and autophagic degradation (Galluzzi et al., 2017; Murthy et al., 2020; Zhang et al., 2021). cGAS promotes autophagy by interacting with the autophagy regulatory protein Beclin-1, and light chain 3 (LC3). This not only enhances cGAS and DNA’s degradation by autophagy but also inhibits the synthesis of cGAMP to prevent the production of type I interferon, thereby reducing cGAS overactivation and prolonging immune stimulation (Liang et al., 2014; Zheng et al., 2021). TBK1-mediated phosphorylation of p62 allows STING to be degraded *via* autophagy, thus driving STING ubiquitination and autophagic degradation and weakening innate immune signal transduction (Prabakaran et al., 2018). In summary, the cGAS–STING pathway induces autophagy, which can modulate the innate immune response by degrading cGAS and STING and eliminating DNA and pathogens from the cytosol. Autophagy is crucial for preventing an excessive immune response and sustained immune stimulation as well as maintaining cellular homeostasis (Zheng et al., 2021).

## 3. cGAS–STING pathway and neuroinflammation

A variety of pathological injuries, such as ischemia, trauma, infection, and toxins, can elicit neuroinflammation, a CNS defensive response that safeguards the brain by eliminating or suppressing pathogens and encouraging tissue repair. However, persistent neuroinflammation can induce secondary injury, which eventually results in progressive neurodegeneration (Simon et al., 2017; Shen et al., 2019; Leng and Edison, 2021). Microglia, the main resident immune cells in the CNS, recognize and respond to a variety of signals to promote immune defense, and STING is mainly expressed in these cells (Li et al., 2020; Paul et al., 2021). Microglia can have either proinflammatory or neuroprotective effects, depending on their activation state. Proinflammatory microglia (M1) secrete damaging cytokines that might worsen brain injury, whereas anti-inflammatory microglia (M2) secret cytokines that can aid in neurological rehabilitation and brain repair (Shi et al., 2022). Furthermore, neurons and astrocytes also produce interferons (Paul et al., 2021). Astrocytes are the most numerous cells in the CNS; they support CNS stability, maintain neuronal activity, and reabsorb neurotransmitters. Under stress, astrocytes can multiply to form reactive astrocytes with various phenotypes and roles. A1 astrocytes tend to inflammatory activation and can cause the death of neurons, whereas A2 astrocytes have protective effects, including a propensity to limit inflammation, upregulate neurotrophic factors, and support neuronal survival (Kwon and Koh, 2020). The ability to regulate the intrinsic mechanisms that govern the swift shift between the detrimental and advantageous phenotypes of microglia and astrocytes may lead to the development of novel treatments for CNS illnesses (Zhao et al., 2021; Shi et al., 2022).

**Figure 2:**
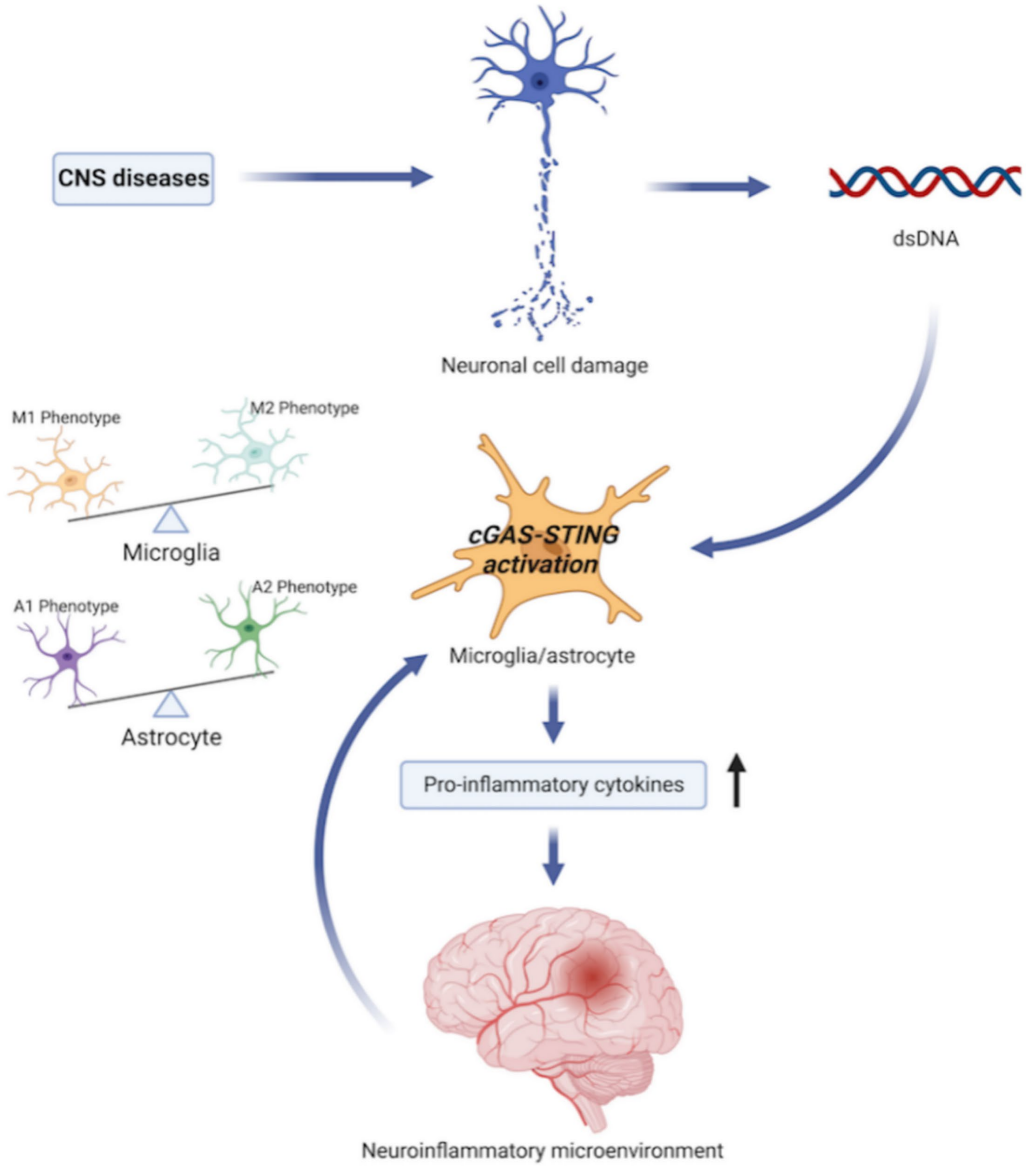
cGAS–STING pathway and neuroinflammation. Central nervous system (CNS) diseases, such as ischemic stroke, neurodegenerative disorders, traumatic brain injury etc., cause neuronal cell damage and releases dsDNA, including cytosolic DNA and mitochondrial DNA. dsDNA acts on neighboring microglia and astrocytes to activate intracellular cGAS–STING signaling pathway. Meanwhile, the activation of cGAS–STING pathway encourages microglia and astrocyte to secret proinflammatory cytokines, and affects the inflammatory phenotype of microglia and astrocytes, which creates a necessary neuroinflammatory microenvironment for the development of CNS diseases. In addition, disruption of neuroinflammatory microenvironment homeostasis may in turn affects the balance of pro-inflammatory and anti-inflammatory phenotypes of microglia and astrocytes.

Reactive oxygen species (ROS) have been linked to neurodegenerative illnesses in many studies. Neuronal oxidative stress contributes to neurodegenerative illnesses by causing internal mitochondrial damage, increasing the production of ROS by respiratory chain complexes, and impairing the integrity of the mitochondrial DNA (mtDNA) produced by internal mitochondria. The damaged mtDNA acts on the adjacent microglia and astrocytes, subsequently activating the intracellular cGAS–STING pathway and encouraging the release of pro-inflammatory molecules, thereby creating a neuroinflammatory microenvironment, which is essential for the development of neurodegenerative disorders. In addition, the disruption of neuroinflammatory microenvironment homeostasis may affect the inflammatory phenotypes of microglia and astrocytes (Zhao et al., 2021). Generally, the cGAS–STING pathway in the brain mediates immune surveillance and has a neuroprotective effect; nevertheless, over participation of this system can result in neuroinflammation and neurodegeneration (Figure 2). One study *in vivo* and *in vitro* observed that microglia cells were quickly activated following ischemic stroke (IS), and the cGAS–STING signaling pathway in the microglia was triggered to encourage the development of a pro-inflammatory microenvironment (Kong et al., 2022). Targeting cGAS–STING to control the proliferation and activation of microglia and regulate the balance between the M1 and M2 phenotypes can control excessive neuroinflammation, improve the prognosis of cerebrovascular diseases, and slow neurological degeneration (Zhao et al., 2021; Kong et al., 2022). However, the specific nerve cells and mechanisms involved in STING-mediated neuroinflammation have not been fully elucidated. STING has a significant impact on neuroinflammation and autophagic dysfunction following TBI, as demonstrated in mice models (Abdullah et al., 2018). After TBI, the STING and IFN-I pathways are co-activated, and astrocytes are the main cells involved in STING-mediated responses after TBI. According to Kong et al., both *in vivo* and *in vitro*, STING is activated and predominantly detected in microglia following IS (Kong et al., 2022). Through downstream pathways, STING stimulates microglia to polarize toward the M1 phenotype and inhibits the polarization of M2 microglia. After cerebral ischemia/reperfusion (I/R) injury, inhibition of STING promotes the microglial phenotype towards the M2 phenotype. STING regulates microglial polarization through the activation of IRF3 and NF-κB pathways. NLRP3 Inflammasome may also play a role in the STING-mediated polarization of microglia (Wang et al., 2020; Xiao et al., 2020). Targeting cGAS–STING may be a promising therapeutic approach for CNS illnesses, but further research is required to understand how it specifically contributes to neuroinflammation.

## 4. Role of the cGAS–STING pathway in neurological disorders

### 4.1. cGAS–STING pathway and ischemic stroke

Ischemic stroke is a serious neurological condition characterized by a temporary or permanent reduction of local cerebral blood flow. Its high morbidity, high associated incidence of disability, and poor prognosis place a substantial societal burden on the global community (Li et al., 2020; Jiang et al., 2021; Kong et al., 2022).

The cGAS–STING pathway has a significant function following ischemic stroke, as evidenced by an increasing number of studies, and modulation of this pathway may play a significant role in the treatment of ischemic stroke. After cerebral ischemia stroke, necrotic neurons release cytoplasmic dsDNA, which is a potential damage-associated molecular pattern (DAMP) that can activate the cGAS–STING pathway. Qian et al. found in a mouse model that the synthetic oligodeoxynucleotide A151, comprised of the immunosuppressive motif TTAGGG as a cGAS inhibitor, ameliorates the inflammatory environment and reduces infarct volume; it also improves long-term outcomes after cerebral infarction by reducing the proliferation and activation of local microglia in the ischemic penumbral zone and decreasing the migration of periphery neutrophils into the CNS (Li et al., 2020).

At the same time, neovascularization and perfusion of ischemic peripheral cerebrovascular structure play important roles in stroke recovery (Hoang et al., 2009; Mostany et al., 2010). Moreover, newly formed blood vessels are not completely developed, and the increased permeability of the vasculature causes the opening of the blood–brain barrier (BBB), leading to increased extravasation of immune cells and toxic proteins from the blood. Therefore, maintaining neovascular stability and restoring the damaged BBB may be critical for the steady brain microenvironment necessary to improve stroke recovery (Yepes et al., 2003; Daneman, 2012; Obermeier et al., 2013; Xu et al., 2017). And regulation of the cGAS–STING pathway may promote neovascularization and lessen BBB damage after IS.

Kang et al. (2020) found that neutrophil extracellular traps (NETs)—extensive web-like DNA structures—were formed after stroke, which induced STING pathway activation. After IS, neutrophils gather in the peri-infarct cortex. When neutrophils are activated, nuclear and granular contents are released, creating NETs. Increased NET creation or reduced NET clearance is not conducive to revascularization and vascular repair, and the inhibition of the type I IFN response contributes to cerebral vascular recovery (Kang et al., 2020). NETs contain dsDNA, histones, and granular proteins, and they releases several cytotoxic proteases, such as neutrophil elastase, cathepsin G, and myeloperoxidase (MPO), which directly cause endothelial cell injury and thus increase vascular permeability (Urban et al., 2009; Villanueva et al., 2011; Kang et al., 2020). The enzyme peptidylarginine deiminase 4 (PAD4), which is upregulated in the peri-ischemic brain, is necessary for NETs production. PAD4 overexpression increases NETs formation, accompanied by decreased neovascularization as well as enhanced BBB injury. The destruction of NETs by deoxyribonuclease 1 (DNase 1) and the suppression of NET production by genetic ablation or drug inhibition of PAD can increase neovascularization and vascular restoration as well as decrease BBB damage, thus enhancing functional recovery after cerebral ischemia in mice. In addition, Kang et al. discovered in a mouse model that PAD inhibition decreases the amount of IFN-β induced by stroke, a process that is mediated by STING; moreover, STING knockdown and IFN receptor-neutralizing antibody therapy decrease BBB disruption and enhance vascular plasticity (Kang et al., 2020).

Currently, thrombolysis with tissue plasminogen activator (tPA) is one of the most important treatments for acute IS. It allows reperfusion of infarct areas, but the increased risk of a cerebral hemorrhage after thrombolytic therapy limits its use in IS (Tanne et al., 2002; Whiteley et al., 2012; Jauch et al., 2013). tPA activates the cerebral endothelium, resulting in the deterioration of vascular integrity and acute disruption of the BBB (Yepes et al., 2003; Suzuki et al., 2016), and it stimulates the recruitment of neutrophils to ischemic tissue (Uhl et al., 2014; Shi et al., 2021). Wang et al. found that tPA promoted the accumulation of neutrophils in ischemic brain tissue, upregulated PAD4, and increased NET formation *in vitro*. NET release impaired cerebral vascular integrity and exacerbated tPA-induced cerebral hemorrhage and acute BBB destruction. Additionally, the authors discovered that the activation of the cGAS–STING pathway and its mediated type I IFN response affected the NET-mediated impact on tPA-related cerebrovascular complications in stroke. After stroke, tPA therapy greatly enhanced the expression of cGAS and the activation of STING in the microglia; it also increased the production of the downstream signals of pTBK1, pIRF3, and IFN-β within the ischemic cortex. It was demonstrated in mice models that DNase I clears NETs, protects BBB integrity, and lessens tPA-related cerebral hemorrhage. In addition, its antihemorrhagic effect is primarily induced by suppressing the activation of the cGAS–STING pathway (Wang et al., 2021). To increase the safety of tPA thrombolytic therapy, a novel strategy that targets NETs or the cGAS–STING pathway may be beneficial. Nevertheless, additional research will be required in the future to identify therapies that can be applied in clinical practice.

Additionally, the deficiency of oxygen and glucose in the local cerebral tissue caused by IS promotes neuroinflammation, neuronal cell death, and secondary tissue damage in I/R (Stevens et al., 2008; Denes et al., 2015; Han et al., 2020). *In vitro* studies have shown that oxygen and glucose deficiency can directly activate microglia and subsequently trigger the IFN I pathway, leading to neuroinflammation, which is detrimental to the prognosis of IS. Histone deacetylases (HDACs) play a role in recovery after stroke and I/R-induced brain damage. Liao et al. reported that HDAC transcriptionally controls cGAS and identified a novel HDAC3–p65–cGAS signaling pathway (Liao et al., 2020). The downregulation of cGAS in microglia contributes to the reduction of neuroinflammation and I/R-induced brain damage. The expression of cGAS is controlled transcriptionally by HDAC3 in a p65-dependent manner. To encourage cGAS–STING signaling activation and neuroinflammation in microglia, HDAC3 deacetylates p65 and increases cGAS expression in transcription (Liao et al., 2020). Therefore, the cGAS–STING pathway can be reduced by deleting HDAC3 or inhibiting HDAC3 in microglia, thus alleviating acute I/R-induced neuroinflammation and cerebral damage. These findings indicate that the HDAC3–p65–cGAS signaling pathway plays a critical role in neuroinflammation and tissue damage caused by IS, representing a promising area for novel IS therapy approaches.

At present, restoring blood reperfusion in the ischemic area (i.e., restoring blood oxygen supply to the brain tissue) is the primary clinical treatment for stroke. Nevertheless, the overactive immune response in the brain following reperfusion typically exacerbates the pathological injury and clinical symptoms of the nervous system, which impact the recovery of patients with IS (Shi et al., 2022). To lessen reperfusion injury and improve the long-term survival rate after stroke, improving the intracerebral immunological environment is critical. The activation of microglia and the surrounding infiltrating inflammatory cells in the brain after stroke leads to a complicated, hyperactive brain immune microenvironment, which represents the primary barrier to neurological repair following IS. Generally, pro-inflammatory microglia (M1), which release cytokines that worsen brain damage, have opposite effects to anti-inflammatory microglia (M2), which release cytokines that reduce neurological impairment and encourage brain recovery (Shi et al., 2022). Therefore, the regulation of microglia phenotypes may contribute to the advancement of new stroke treatments. Jiang et al. created an *in vitro* oxygen–glucose deprivation (OGD) cell model using HT22 cells and subsequently used the cell culture supernatants that contained OGD-induced DAMPs (OIDs) to stimulate BV2 microglia. They found that the polarization of microglia was closely related to the cGAS–STING signaling pathway. By suppressing the activation of the cGAS–STING pathway, the downregulation of cGAS significantly lowered the release of inflammatory molecules to promote M2 microglia polarization, reduce neuroinflammation, and improve neurological dysfunction (Jiang et al., 2021). In another study aiming to manage the hyperactive cerebral immune microenvironment, Shi et al. (2022) reported an engineering CXCL12 biomimetic decoy-integrated versatile immunosuppressive nanoparticle (VIN). The VIN was prepared by coating CXCR4-rich mesenchymal stem cells (MSCs) membrane vesicles onto A151-loaded polydopamine nanospheres (PDA). Loaded A151 inhibited the cGAS–STING pathway in microglia, causing microglia to polarize into the M2-like phenotype and play an anti-inflammatory role. The bridge of Zn^2+^ effectively allowed A151 to be loaded onto PDA. Within the inflammatory region, PDA was oxidized by ROS with the loss of the Zn^2+^ complexation effect, and subsequently, A151 was released, allowing accurate drug delivery and controlled release in the brain. In the rat IS model, the VIN has the effects of peripheral inflammatory cell filtration, polarization of activated microglia in the brain, and scavenging ROS, thus improving the brain immune microenvironment, protecting neurons, reducing the infarct volume, relieving brain damage, and decreasing mortality (Shi et al., 2022). With these benefits, VIN is expected to become a new IS treatment. STING was activated and primarily detected in microglia following IS, according to research by Kong et al. who employed an *In vivo* middle cerebral artery occlusion (MCAO) model using adult male C57BL/6 mice and an *in vitro* oxygen–glucose deprivation/reperfusion (OGD/R) paradigm using BV2 microglia (Kong et al., 2022). The inhibition of STING significantly decreased the number of M1 microglia and promoted the transition of microglia to the M2 phenotype after brain I/R injury. A common method of blocking STING is the use of C-176, a highly effective and selective small molecule antagonist of STING (Kong et al., 2022). This method significantly reduced cerebral infarction size, brain edema, neuronal apoptosis, and degeneration, thereby restoring neurological function at different stages and improving stroke outcomes in mice models. Taken together, targeting the cGAS–STING pathway promotes neovascularization and damaged BBB restoration, lessens I/R-induced brain injury, and modulates microglial phenotype to improve the intracerebral immunological environment after IS. While additional research is required to fully understand the precise mechanism of action of the cGAS–STING pathway following IS, regulation of this pathway is anticipated to be a novel therapeutic approach for the condition.

### 4.2. cGAS–STING pathway and neurodegenerative disorders

Alzheimer’s disease (AD), Parkinson’s disease (PD), Huntington’s disease (HD), amyotrophic lateral sclerosis (ALS), multiple sclerosis (MS), ataxia–telangiectasia (A–T), and other neurodegenerative disorders are uncommon genetic diseases of the CNS that result in a slow and gradual loss of function of certain groups of neurons and their connections. Increasing studies have revealed that the cGAS–STING pathway plays a significant role in the development of neurodegenerative diseases. Neuronal oxidative stress contributes to neurodegenerative illnesses by damaging internal mitochondria and increasing the generation of ROS by the respiratory chain complex, which subsequently leads to damage to the integrity of mtDNA released by internal mitochondria. mtDNA release caused by ROS accumulates in the cytoplasm, acts on the microglia and astrocytes of neighboring neurons, and ultimately activates the intracellular cGAS–STING pathway. The activated cGAS–STING pathway stimulates microglia and astrocytes to secrete pro-inflammatory molecules, and affects the inflammatory phenotypes of neighboring microglia and astrocytes, which maintain the neuroinflammatory microenvironment necessary for neurodegenerative disorders (Zhao et al., 2021). Neuroinflammation and neurodegeneration are exacerbated by the overactivation of the cGAS–STING pathway in the brain. A potential approach to treating neurodegenerative illnesses involves regulating the mtDNA–cGAS–STING pathway to reverse the phenotypic changes in microglia and astroglia. Although various therapeutic approaches to the cGAS–STING pathway have been discovered so far, they are still in the stage of animal and cell experiments, and additional research is required to investigate the therapeutic approaches used in clinical practice in the future. For example, melatonin is a neuroprotective hormone that effectively reduces free radicals. Jauhari et al. (2020) demonstrated that melatonin deficiency increased mitochondrial ROS injury and subsequent mtDNA release with the activation of cGAS, resulting in pathological neuroinflammation; by contrast, melatonin supplementation had a neuroprotective effect in mice models. This finding may provide a new therapeutic direction for neurodegenerative diseases. Additionally, Kwon et al. demonstrated that the inhibition of glial serum/glucocorticoid-related kinase 1 (SGK1) blocked the intracellular inflammatory pathways mediated by cGAS–STING and NF-κB, thereby regulating the pro-inflammatory characteristics of glial cells (Kwon et al., 2021). Moreover, the suppression of SGK1 enhanced glial activity to eliminate glutamate toxicity and reduce glial cell senescence and mitochondrial damage (Kwon et al., 2021); this could represent a novel treatment strategy for PD, AD, and other neurodegenerative disorders associated with glial cell-mediated neuroinflammation. What’ more, Mathur et al. found that the antiviral drug ganciclovir (GCV), especially the GCV dimer, activated the IFN-I response in a STING-dependent manner and reduced microglia proliferation and neuroinflammation *In vivo*, suggesting the potential for the creation of a new group of medications to cure diseases associated with neurodegeneration and neuroinflammation (Mathur et al., 2017).

It has been shown that the primary risk factor for most neurodegenerative illnesses is aging, which is related to neuroinflammation and the buildup of senescent cells (Herbig et al., 2006; van Deursen, 2014). Cellular senescence is a condition of replication stagnation that results in sustained chronic low-grade inflammation in the brain accompanied by neurodegeneration and decreased neuronal plasticity, ultimately leading to cognitive decline and deficits (Bektas et al., 2018; Franceschi et al., 2018). The complicated combination of pro-inflammatory cytokines, chemokines, growth factors, and proteases secreted by senescent cells is known as the senescence-associated secretory phenotype (SASP). SASP may lead to tissue injury and long-lasting chronic inflammation, thereby accelerating the degeneration associated with aging. Recent evidence showed that the cGAS–STING pathway recognizes cytoplasmic chromatin fragments (CCFs) extruded from the nucleus of senescent cells to stimulate SASP (Vizioli et al., 2020). The elimination of senescent cells may have therapeutic benefits, and autophagy is one of the pathways that can remove injured or senescent cells. In addition to accelerating the clearance of CCFs, activated STING, and other cell debris, autophagy can reduce the cytoplasmic DNA load and inhibit SASP, providing new therapeutic directions for neurodegenerative diseases (Han et al., 2020).

In summary, targeting the cGAS–STING pathway offers a novel therapeutic strategy for neurodegenerative diseases, while stimulating autophagy to eliminate senescent cells and activated STING can also be employed as a new treatment. However, there is a lack of effective treatments applied in clinical practice.

#### 4.2.1. Alzheimer’s disease

Alzheimer’s disease is a progressive and deadly neurodegeneration disease marked by gradual impairment of cognitive function; currently, no viable therapeutic approaches are available. Previous studies have shown that neuroinflammation, mitochondrial dysfunction, and cellular senescence play key roles in the development of AD (Johri and Beal, 2012; Heneka et al., 2015; Bussian et al., 2018; Fu et al., 2019). Moreover, defects in mitophagy in AD samples lead to the excessive release of DNA into the cytoplasm, which increases the activation of the cGAS–STING pathway and results in aberrant neuroinflammation and cellular senescence. Previous research has demonstrated the detrimental effects of IFN-I signaling in hippocampal neurogenesis and brain function in response to aging, which is the biggest risk factor for AD (Baruch et al., 2014; Taylor et al., 2018). Reducing IFN-I-associated neuroinflammation may alleviate the progression of AD. Furthermore, Nicotinamide adenine dinucleotide (NAD^+^), a crucial metabolite in human cells, is essential for several processes involving mitophagy and DNA repair, both of which are compromised in AD neuronal cells. Hou et al. found that supplementation with the NAD^+^ precursor nicotinamide riboside (NR) could stimulate mitophagy to reduce cytoplasmic DNA and the cGAS–STING signaling, thereby decreasing neuroinflammation and cellular senescence in the brains of AD mice (Hou et al., 2021). In addition, microglia and astrocytes in the AD brain activated and released inflammatory cytokines that promoted neurodegeneration, whereas NR switched the microglia from a deleterious to a protective phenotype (Hou et al., 2021). In conclusion, supplementation of NR to modulate cGAS–STING signaling and reducing IFN-I-associated neuroinflammation may provide a new direction for the treatment of AD.

#### 4.2.2. Parkinson’s disease

Parkinson’s disease is a common progressive CNS disease marked by degeneration of dopamine (DA) neurons in the substantia nigra (SN) of the midbrain and toxic intraneuronal inclusion of misfolded synuclein alpha (Lewy bodies and neurites; Kwon et al., 2021). Its clinical manifestations include tremor, bradykinesia, and rigidity.

A major gene mutation in familial and sporadic PD occurs in leucine-rich repeat kinase 2 (*LRRK2*). In LRRK2-knockout macrophages, increased mitochondrial fission induced by dynamin-related protein (Drp1) and enhanced oxidative stress lead to mtDNA release and chronic activation of the cGAS–STING pathway. The inhibition of Drp1 and antioxidant treatment in LRRK2 knockout macrophages can alleviate this stress (Weindel et al., 2020). Furthermore, mutations in PINK1 and Parkin contribute to early-onset Parkinson’s disease. Recent studies have indicated that Parkin and PINK1-deficient mice have defects in mitophagy, which results in the release of mtDNA and the activation of the cGAS–STING pathway (Borsche et al., 2020). Treatments targeting cGAS–STING and the stimulation of mitophagy can be used in new therapeutic approaches.

#### 4.2.3. Huntington’s disease

Huntington’s disease is an autosomal dominant progressive nervous system disorder brought on by glutamine expansion in the huntingtin protein (HTT), resulting in muscle wasting, motor and cognitive impairments, psychiatric disorders, and neurodegeneration (Bates et al., 2015). HD progresses because of the widespread expression of the mutant gene *mHTT*, which causes significant injury to the striatum and cortex and contributes to extensive peripheral impairments as the disease develops (Sharma et al., 2020). This affects fundamental cellular processes, like transcriptional regulation, DNA repair, and nucleoplasmic transport (Shirasaki et al., 2012), thereby increasing ROS damage, mtDNA release, cGAS pathway activation, and pathological inflammatory responses; ultimately, this results in the synaptic loss and neurodegeneration (Jauhari et al., 2020).

An HD cell culture model demonstrated that cytosolic mtDNA is elevated in HD, and transfection of DNase I into these cells reduced inflammation. Melatonin functions as an effective free radical scavenger that inhibits mHTT-mediated neurotoxicity in mouse models of HD (Jauhari et al., 2020). In another study, Sharma et al. (2020) found that cGAS is upregulated in HD and mediates neuroinflammation and autophagic responses in HD cells. Therefore, targeting the cGAS–STING pathway is a promising direction to treat HD.

#### 4.2.4. Amyotrophic lateral sclerosis

ALS, also known as motor neuron disorder, is an adult-onset neurodegenerative disease marked by the progressive loss of upper and lower motor neurons, which results in muscle weakness, severe disability, and, ultimately, paralysis (Swarup et al., 2011; Hardiman et al., 2017). Numerous studies have shown that neuroinflammatory responses are involved in the pathogenesis of the disease. Transactive response DNA-binding protein 43 (TDP-43) is a multifunctional nucleic acid-binding protein, and its inclusions are a hallmark of ALS. ALS-associated TDP-43 mutations mediate microglial activation and trigger the activation of NF-κB and the production of pro-inflammatory factors, causing a pro-inflammatory cascade that is deleterious to motor neurons (Swarup et al., 2011; Zhao et al., 2015). Recently, Yu et al. discovered that TDP-43 triggers the release of mtDNA into the cytoplasm in mice, thus activating the cGAS–STING pathway and leading to inflammation in ALS (Yu et al., 2020). In conclusion, targeting the cGAS–STING pathway may contribute to the development of novel therapeutic approaches for ALS.

#### 4.2.5. Multiple sclerosis

Multiple sclerosis is a chronic inflammatory illness of the CNS that lacks viable treatment options. MS is characterized by glial activation, inflammatory cell infiltration, nerve fiber demyelination, and BBB disturbances (Reich et al., 2018).

Mathur et al. (2017) employed experimental autoimmune encephalitis (EAE) as a model for MS and discovered that STING is crucial for improving IFN-I-mediated neuroinflammation. Furthermore, IFN-I plays a protective role in MS. The antiviral drug GCV, which has received Food and Drug administration (FDA) approval, activates the IFN-I response and reduces microglial proliferation and inflammation of the nervous system *in vivo* in a STING-dependent manner (Mathur et al., 2017). Another study in mice found an inverse association between ultraviolet (UV) radiation and the incidence of MS (Hart et al., 2011; Sontheimer et al., 2017). UVB irradiation could recruit inflammatory monocytes and trigger the production of IFN-I *via* a STING-dependent mechanism. Considering the aforementioned findings, regulating the cGAS–STING pathway to activate IFN-I response may contribute to developing new treatments for MS.

#### 4.2.6. Ataxia–telangiectasia

A–T is an inherited disease brought on by homozygous or compound heterozygous mutations in the ataxia–telangiectasia mutated gene (*ATM*), which encodes ATM kinase. Patients with A–T exhibit enhanced genome instability, altered nuclear shape, accumulation of micronuclei, neuronal impairments, and premature entry into cellular senescence; together, these contribute to ataxia, immunodeficiency, cancer susceptibility, neurodegeneration, and premature aging.

Senescence-associated secretory phenotype, secreted by senescent cells, is a key factor in the promotion of neurological defects. Using human pluripotent stem cell-derived cortical brain organoids, Aguado et al. found that the suppression of cGAS–STING significantly inhibited the expression of SASP triggered by self-DNA in A–T brain organoids, suppressed astrocyte senescence and neurodegeneration, and improved the neuropathology of A–T brain organoids (Aguado et al., 2021). In patients with A–T, overactivation of cGAS–STING is a major contributor to chronic inflammation and premature aging. The cGAS inhibitor aspirin and STING inhibitor H151 effectively inhibit the cGAS–STING pathway, and they suppress senescent astrocyte-driven inflammation in related brain organoid models (Haag et al., 2018; Dai et al., 2019; Aguado et al., 2021). In summary, inhibiting the cGAS–STING pathway is an effective therapeutic approach for A–T and may be useful in other neurological conditions linked to premature aging and self-DNA-induced activation of SASP.

### 4.3. cGAS–STING pathway and herpes simplex virus encephalitis

Herpes simplex virus (HSV) type 1, a neurotropic virus, is a leading cause of CNS infections involving herpes simplex virus encephalitis (HSE), which may cause irreversible damage to the CNS. Innate immune responses are critical for controlling HSV-1 in the CNS, and IFN-I is critical for these responses. Reinert et al. identified microglia as a major producer of IFN-I after HSV-1 infection; the cGAS–STING pathway was used to stimulate this response (Reinert et al., 2016). HSV-1 belongs to the Alphaherpesvirus subfamily and has a double-stranded linear DNA genome (Huang et al., 2018); during HSV-1 replication, viral DNA is released into the cytoplasm. Moreover, studies have demonstrated that HSV-1 infection can induce mitochondrial stress, and mtDNA is also released into the cytoplasm. cGAS can sense DNA leaking into the cytoplasm and stimulate the cGAS–STING pathway, resulting in activation of IRF3 and NF-κB, followed by the production of interferons and inflammatory chemokines to inhibit HSV-1 replication (Xu et al., 2017; Huang et al., 2018). IRF3 is a crucial transcription factor in the IFN-β signaling pathway, and the phosphorylation and dimerization of IRF3 are markers of early IFN-mediated antiviral response (Huang et al., 2018). In addition, the cGAS–STING pathway is strictly regulated by post-translational modifications, including phosphorylation, palmitoylation, amidation, glutamylation, and ubiquitination (Bodda et al., 2020).

To successfully infect the host, HSV-1 can suppress cGAS–STING through several different viral proteins, thereby counteracting the host immune response, mediating immune evasion, and establishing lifelong latent infection (Zhang et al., 2016). Understanding the mechanisms by which HSV-1 evades host antiviral defense may elucidate novel targets for the development of anti-HSV-1 therapies (Table 1).

**Table 1:** Different viral proteins to suppress cGAS-stimulator of interferon genes (STING) pathway.

Viral protein	Function	References
VP1-2	Deubiquitinates STING	Bodda et al. (2020)
VP16	Binds to p65 to inhibit NF-κB activation	Xing et al. (2013)
VP22	Inhibits the enzymatic activity of cGAS	Huang et al. (2018)
VP24	Inhibits the phosphorylation and dimerization of IRF3	Zhang et al. (2016)
ICP0	Binds to p65/p50 to inhibit NF-κB activation	Zhang et al. (2013a)
ICP27	Binds to IκBα to inhibit NF-κB activation	Kim et al. (2008)
US3	hyperphosphorylated p65 to inhibit NF-κB activation	Wang et al. (2014)
UL24	Reduces the nuclear translocation of p65/p50 to inhibit NF-κB activation	Xu et al. (2017)
UL42	Binds to p65/p50 to inhibit NF-κB activation	Zhang et al. (2013b)
UL37	Inhibits the activation of cGAS	Zhang et al. (2018)
UL41	Decreases cGAS expression	Su and Zheng (2017)
UL46	Reduces TBK1 activation	You et al. (2019)

STING, stimulator of interferon genes; cGAS, cyclic GMP–AMP synthase; IRF3, interferon regulatory factor 3; NF-κB, nuclear factor-κappa B; IκBα, inhibitor of NF-κB; TBK1, TANK-binding kinase 1.

### 4.4. cGAS–STING pathway and traumatic brain injury

Traumatic brain injury (TBI) is a major global cause of adolescent death and permanent disability (Lingsma et al., 2010); it is characterized by initial irreversible damage at the site of impact, followed by delayed secondary molecular and cellular damage responses, including neuronal cell death, initiation of resident brain immune cells (mainly microglia and astrocytes) and infiltration of peripheral leukocytes and a subsequent release of inflammatory cytokines, chemokines, and other secondary messengers, leading to chronic progressive neurodegenerative changes (Loane et al., 2014; Abdullah et al., 2018; Barrett et al., 2020). DAMP, like cytosolic DNA and mtDNA, is produced from damaged neurons after CNS injury, triggering innate immune signaling, including cGAS–STING, which activates glial cells and promotes secondary neuroinflammation (Walko et al., 2014; Chin, 2019; Wang et al., 2019). Following TBI, increased activation of the cGAS–STING and IFN-I signaling is linked to severe neuroinflammation. Inhibiting cGAS–STING in animal models has been demonstrated to decrease neuroinflammation and improve functional recovery following TBI. And the positive effect of suppressing cGAS–STING was related to a decrease in IFN-I production and signaling (Karve et al., 2016; Abdullah et al., 2018; Barrett et al., 2021). Additionally, evidence suggests that markers of autophagy increase following TBI, leading to both protective and deleterious effects. Persistent neuroinflammation and autophagic dysfunction contribute to cellular injury and neurological deficits after TBI (Abdullah et al., 2018).

According to research by Barrett et al., IFN-β promotes secondary neuroinflammation after TBI. Suppressing IFN-β decreases post-traumatic neuroinflammation and neurodegeneration, which improves long-term motor and cognitive repair (Barrett et al., 2020).

In another study in mice, Abdullah et al. found that STING had a deleterious effect in mediating TBI-induced neuroinflammatory responses and autophagic dysfunction; the STING and type I IFN pathways were co-activated after TBI, both of which are critical factors that exacerbate TBI injury outcomes (Abdullah et al., 2018). As a protective mechanism to reduce cellular damage, autophagic activity is enhanced following TBI; however, STING may be a critical regulator of autophagic dysfunction after TBI. In summary, targeting cGAS–STING to reduce IFN-I production and modulate autophagy may serve as a novel therapeutic target to reduce injury following TBI.

### 4.5. cGAS–STING pathway and other CNS disorders

Aicardi–Goutières syndrome (AGS) is an autosomal recessive inflammatory neurological disease marked by intellectual disability, microcephaly, immune disorders, and childhood mortality. It is brought on by mutations in any of the following genes: *TREX1*, *RNASEH2A*, *RNASEH2B*, *RNASEH2C*, *SAMHD1*, *ADAR*, and *IFIH1* (Crow et al., 2015). TREX1 (DNase III) is a major 3′ DNA exonuclease responsible for cytosolic DNA degradation (Morita et al., 2004), and TREX1 deficiency in the cytoplasm of patients with AGS leads to the accumulation of cytoplasmic DNA, which drives chronic inflammation caused by cGAS.

Liu et al. (2019) found that GTPase-activating protein SH3 domain-binding protein 1 (G3BP1, a protein known to regulate the RNA stress response) initiates cGAS to achieve effective DNA binding, and the targeted inhibition of G3BP1 could treat cGAS-mediated chronic inflammation in the cells of patients with AGS. A natural chemical found in green tea called epigallocatechin gallate (EGCG) selectively targets and inhibits G3BP1, significantly suppressing DNA-induced cGAS activation and subsequent IFN-I production; thus, it plays an important role in the treatment of disorders linked to cGAS (Liu et al., 2019). In addition, Dai et al. (2019) demonstrated that aspirin, a non-steroidal anti-inflammatory medicine, could inhibit cGAS activation and suppress its mediated immune responses through acetylation in AGS patient cells and in an AGS mouse model; therefore, it can be used to treat AGS and other DNA-mediated autoimmune diseases. In conclusion, modifying the cGAS–STING pathway and its downstream signaling molecules is expected to be an effective approach for the treatment of AGS (Table 2).

**Table 2:** Therapeutic approaches targeting cGAS-STING signaling pathway for central nervous system (CNS) diseases.

CNS diseases	Therapeutic approaches	Mechanism	Type of study	References
IS	A151	Inhibits cGAS	*In vivo* and *in vitro* model	Li et al. (2020)
	HDAC3 inhibitors	Inhibits cGAS	*In vivo* and *in vitro* models	Liao et al. (2020)
	VIN	Inhibits cGAS	*In vivo* and *in vitro* models	Shi et al. (2022)
	C-176	Inhibits STING	*In vivo* and *in vitro* models	Kong et al. (2022)
AD	NR	Stimulates mitophagy, reduce cytoplasmic DNA	*In vivo* and *in vitro* models	Hou et al. (2021)
PD	Inhibits Drp1	Decreases mitochondrial fission, reduce mtDNA	*In vivo* and *in vitro* models	Weindel et al. (2020)
HD	Melatonin	Decreases mitochondrial ROS injury, reduce mtDNA	*In vivo* and *in vitro* models	Jauhari et al. (2020)
MS	GCV	Activates the IFN-I response in a STING-dependent manner and reduces neuroinflammation	*In vitro* models	Mathur et al. (2017)
A-T	H-151	Inhibits STING	*In vitro* models	Aguado et al. (2021)
	Aspirin	Inhibits cGAS through acetylation	*In vitro* models	Aguado et al. (2021)
AGS	EGCG	Inhibits G3BP1 to suppresses the activation of cGAS	*In vivo* and *in vitro* models	Liu et al. (2019)
	Aspirin	Acetylates cGAS to inhibit cGAS-mediated immune responses	*In vivo* and *in vitro* models	Dai et al. (2019)

A151, synthetic oligodeoxynucleotide; cGAS, cyclic GMP–AMP synthase; HDAC3, histone deacetylase 3; VIN, versatile immunosuppressive nanoparticle; C-176, a small molecule inhibitor of STING; STING, stimulator of interferon genes; AD, Alzheimer’s disease; NR, nicotinamide riboside; PD, Parkinson’s disease; Drp1, dynamin-related protein; mtDNA, mitochondrial DNA; HD, Huntington’s disease; MS, multiple sclerosis; GCV, Ganciclovir; IFN-I, type I interferon; A-T, ataxia–telangiectasia; H-151, a small molecule inhibitor of STING; AGS, Aicardi-Goutières syndrome; EGCG, epigallocatechin gallate; G3BP1, GTPase-activating protein SH3 domain–binding protein 1.

## 5. Conclusion

The cGAS–STING pathway acts as an innate immune sensor that recognizes pathogen-derived DNA and self-DNA, resulting in the activation of inflammatory signaling pathways. Although normal CNS function requires the involvement of the immune system, persistent and excessive immune stimulation can be detrimental. The cGAS–STING pathway is a double-edged sword, and regulating this pathway can be helpful for the treatment of CNS diseases. This review described the mechanism of action of cGAS–STING in CNS diseases and summarized the current therapeutic targets of promising interventions. However, at present, the research on the treatment of CNS-related diseases by targeting the cGAS–STING pathway is still in the stage of animal and cell experiments while human clinical trials have been disappointing; many more studies are urgently needed to identify interventions that precisely act on this pathway and its upstream and downstream factors and explore therapeutic methods applied in clinical practice.

## Author contributions

JD, YD, and JZ performed the literature review and wrote the manuscript. HZ and BT helped with the outline and article modification. All authors contributed to the article and approved the submitted version.

## Funding

The work was supported by Zhejiang Provincial Medical and Health Technology Project for Young Backbone Talents (Grant No. 2019RC234).

## Conflict of interest

The authors declare that the research was conducted in the absence of any commercial or financial relationships that could be construed as a potential conflict of interest.

## Publisher’s note

All claims expressed in this article are solely those of the authors and do not necessarily represent those of their affiliated organizations, or those of the publisher, the editors and the reviewers. Any product that may be evaluated in this article, or claim that may be made by its manufacturer, is not guaranteed or endorsed by the publisher.
